# Book Reviews

**Published:** 1909-02

**Authors:** 


### BOOK REVIEWS

A SYSTEM OF SYPHILIS. In six volumes, edited by D'Arcy Power, M. B, Oxon., F. R. C. S. and J. Keogh Murphy, M. D., M. C. Cantab, F. R. C. S., with an introduction by Jonathan Hutchinson, F. R. S. Volume I, Introduction, History, Microbiology, General Pathology; Early Manifestations in Male and Female, and Congenital Syphilis. Oxford University Press, New York and London. Price for set, $81.00, single volumes, $13.50.

This monumental work in six volumes devoted to syphilis, and dedicated to Schaudinn, Fournier, Hoffmann and Hutchinson, is an example of the magnitude of medicine today and of the thorough manner in which medical literature covers the various subjects.

The time for the appearance of this work is particularly opportune, as the discovery of the spirochaeta pallida has wrought such remarkable changes in many of our views of the pathology, to have a work comprehensive in all its details when viewed from the new standpoint makes it exceedingly important to possess such a work as the one we now have under review.

The readable introduction by Hutchinson touches briefly upon the important and mooted points concerning syphilis, and suggests lines for much profitable thought and investigation. Bloch in discussing the origin of syphilis presents much evidence in favor of the view that this disease was carried to Europe by the crew of Christophe- Columbus.

The microbiology of syphilis is treated upon by Elie Metchnikoff, who has taken such an active part in establishing the spirochaeta pallida as the true cause of syphilis, by the inoculation of anthroproid apes and chimpanzees, which thus opened a wide field for useful experiemntation; photomicrographs of the spirochaeta pallida stained in smears in tissue and shown by reflected light are presented in excellent cuts.

The general pathology of syphilis written by F. W. Andrews may truly be said to be a masterpiece of medical literature; his clearness of diction and fascinating style make one loath to lay aside the work. Space forbids the mention that should also be given to the chapters by Lampkin, Shillitoe and Still.

The splendid colored plates deserve especial commendation as also does the translation of certain chapters and binding. So interested has the reviewer been in reading this volume that it will be with impatience that he awaits the appearance of subsequent volumes.

E. G. BALLENGER, M. D.

---

DISEASES OF THE SKIN AND THE ERUPTIVE FEVERS. By Jay Frank Schamberg, A. B., M. D., Prof. of Dermatology and Infections Eruptive Diseases in the Philadelphia Polyclinic and College for Graduates in Medicine, etc. Fully illustrated. Published by W. R. Saunders Co., Philadelphia. Price, $3.00 net.

There is no branch of medicine which presents so many puzzling features to the general practitioner as diseases of the skin. We do not believe there is a recent book on dermatology which affords the physician, however, a more excellent and concise discussion of the diagnosis and treatment of these diseases than does Schomberg's latest work. While the section on Eruptive Fevers has received especial attention, the cutaneous manifestations of all disases of the skin are considered; the work is really a concise one on dermatology including the latest advances in actinotherapy, rontgentherapy and studies in radium.

The book is attractively and substantially bound and contains 204 illustrations, which add much interest to the work. Considered as a medium size book on Diseases of the Skin the reviewer thinks the book deserves the highest commendation.

---

THE CHANGING VALUES OF ENGLISH SPEECH. By Ralcy Husted Bell, published by Hinds, Noble and Eldredge, New York. Price, $1.25, 304 pages.

Dr. Bell has a most fascinating style of expressing himself, both in prose and in verse and when he combines the poetic and artistic, as he has done in the above work, we think it well worth while for any one caring in the least for good English to read and reread this book, both for its subject matter and for its quality as a piece of beautiful literature. He is quite hostile in his criticism of the reformer of our spelling; he says: "the trouble with these expounders is, that their learing confuses rather than clarifies their understanding. Over specialized endeavor often distorts the perspective." He thinks the changes are natural and evolutionary in character and that they are not to be done by the wholesale.

The writer does not remember ever to have read more beautiful poetic prose than much of the writing of Dr. Bell, who formerly was a practitioner even as we.

---

DISEASES OF THE SKIN. By A. H. Ohmann-Dumesinil, A. M., M. E., M. D., Ph. D., etc. Formerly professor of Dermatology and Syphilology in the St. Louis College for Medical Practitioners, the St. Louis College of Physicians and Surgeons, etc. Third edition thoroughly revised and enlarged, 140 illustrations. C. V. Mosby, Medical Book and Publishing Co., St. Louis, Mo.

The author states in his preface that his intention has been to make of this book a pracical guide to the easy recognition of the skin diseases, as well as of their successful treatment. There are ten chapters and an appendix containing two on diet in skin diseases and on food eruptions. Dr. Ohmann-Dnmerinil has succeeded well in carrying out his intention and has presented in a clear, concise manner just the information most desired by students and general practitioners. Numerous prescriptions are given in such a manner as to make it exceedingly easy to apply the treatment recommended. All of the illustrations are good and some of them of unusual interest. We can commend it as a practical book for practical doctors.

---

SURGICAL MEMORIES, by James G. Mumford, M. D., Instructor in Surgery, Harvard Medical School; Visiting Surgeon to the Massachusetts General Hospital; Fellow of the American Medical Association. Illustrated, $2.50, net. Moffat, York & Co., New York.

In this column Dr. Mumford has colleceted many of his interesting papers and addresses, and has added some material hitherto unpublished. The first essay is a narrative sketch of the history of surgery, and embraces accounts of the great heroes of that art; Hippocrates, Galen, Vesalius, Pare, Haller, John Hunter and Lister receive much attention in a philosophical and scholarly manner. Then follows a paper summing up ancient surgical accomplishments; succeeded by biographical essays on Cooper, Brodie, J. C. Warren and Bigelow. The remaining papers are short essays; accounts of special American achievements in medicine; a critical and historical essay on aneurysm; addresses to nurses, and short papers on ethics and on medical education. Altigether Dr. Mumford has produced a book of much interest to those who care to know something of the history of surgery, and who of us does not?

---

PATHOGENIC MICRO-ORGANISMS INCLUDING BACTERIA AND PROTOZOA. A practical manual for students, physicians and health officers, by William H. Park, M. D., Prof. of Bacteriology and Hygiene, University and Belleview Hospital Medical College, New York, etc. Assisted by Anna W. Williams, M. D., Assistant Director of the New York Research Laboratory. Third edition, enlarged and thoroughly revised, with 176 engravings and 5 full page plates. Lea & Febiger, Philadelphia.

The above work is characterized by clearness and practicability; combined with these one finds that the work is thoroughly up-to-date as is shown by the chapters on opsonins and the spirochaetae. It seems eminently suitable to the student and practitioner.

Part I is devoted to the principles of bacteriology, and gives concisely the well known facts, as well as discusses much that is new in this subject, viz: The antagnonism between our bodies and micro-organisms; the nature of the protective defences of the body—Ehrlich's "side chain" and other theories; agglutenotion, etc.

Part II includes bacteria pathogenic to man, individually considered; bacteriology of milk and water.

Part III is given to a modern discussion of Protozoa. Few branches of medicine have made more rapid progress in recent times than has this subject; these advances are clearly reflected and concisely stated in this part of the work. The book concludes with a glossary defining the many new words that have arisen from the development of bacteriology and minimity; the fact that many of these words are not given except in the most recently published dictionaries makes it a distinctly useful feature.

---

A TEXT-BOOK OF HUMAN PHYSIOLOGY, theoretical and practical, by George V. N. Dearborn, A. M., (Harv.), Ph. D., M. D., Prof. of Physiology in the Medical and Dental Schools of Tuft's College, Boston, etc. Illustrated with 300 engravings and 9 plates. Lea & Febiger, Philadelphia.

This book was written primarily for medical and dental practitioners and students and will undoubtedly be warmly welcomed by them, especially the students who have recommended to them by their professors the most enormous, verbose, and complete books that can be obtained; instead of aiding him these voluminous works only bewilder the poor overworked student and often so discourage him that he will not wade through all the detail of experimentation and theories for the few facts which would enable him to pass reasonable examinations and to assist him in his subsequent professional work. If there is one subject needing reforming in our medical colleges it is this question of selecting suitable text-books; they should be suggested with the student's meager knowledge and limited time in view rather than as a pedantic display of one's familiarity with and commendation of the voluminous works which, while most excellent, are not suitable to the student.

This work apparently presents the essential details and the principles of physiology in an attractive form and while not as simplified as might be, is a distinct improvement over some of the works now being recommended for students. The many, and excellent illustrations assist greatly in presenting clearly the subject matter. We heartily commend this book and would like to see a set of books, for students, more like it.

The author acknowledges his great indebtedness to some of his colleagues and students in the preparation of the text and illustrations, but rather stingily refrains from mentioning their names.

There is less likelihood of injuring the deeper vessels in excising tonsils if the instrument is pressed in deeply to engage the organ rather than exerting pressure from the outside.—*American Journal of Surgery.*

---

An hypertrophied lingual tonsil sometimes causes much discomfort, giving a heavy, sore feeling to the base of the tongue. It may be necessary to remove it.—*American Journal of Surgery.*

---

Hard tonsils preponderating in connective tissue, are better removed by the cold snare than by a sharp instrument. The snare closes the blood nerves; the tonsillitome opens them—*American Journal of Surgery.*

---

The differentiation between a specific and tuberculous ulcer of the fauces is sometimes very difficult. As a rule the specific ulcer is shallow, grayish, with a regular margin, not very tender and does not cause dysphagia; on the other hand, a tuberculous ulcer is deeper, more sloughy, irregular in outline, has an outer inflammatory zone, is exquisitively tender and causes great pain on swallowing; laryngeal examination may reveal a tuberculous condition of the cords.—*American Journal of Surgery.*

Supurating arthritides do not always require exposure of the joint or even large incisions, irrigation and drainage. Such treatment invites mixed infection and ankylosis. If the pus be very thin—even though of streptococcic origin—thorough *aspiration* (which may need to be repeated) and immobilization may effect a rapid cure with perfect function. Purulent arthritis and periarthritis as it occurs in small children as a complication of one of the exanthemata (often in connection with trauma) is often quite amenable to conservative, and even ambulant treatment; aspiration, or irrigation and drainage, and immobilization. Judgment is needed, of course, to determine what cases are amenable to this conservative surgery, and what point in the treatment it must be abandoned in favor of more extensive intervention.—*American Journal of Surgery.*

